# ^13^[C]-Urea Breath Test as a Novel Point-of-Care Biomarker for Tuberculosis Treatment and Diagnosis

**DOI:** 10.1371/journal.pone.0012451

**Published:** 2010-08-27

**Authors:** Mandeep S. Jassal, Gueno G. Nedeltchev, Jong-Hee Lee, Seong Won Choi, Viorel Atudorei, Zachary D. Sharp, Vojo Deretic, Graham S. Timmins, William R. Bishai

**Affiliations:** 1 Center for Tuberculosis Research, Johns Hopkins University School of Medicine, Baltimore, Maryland, United States of America; 2 Division of Pediatric Pulmonology, Johns Hopkins University School of Medicine, Baltimore, Maryland, United States of America; 3 Division of Pharmaceutical Sciences, University of New Mexico, Albuquerque, New Mexico, United States of America; 4 Department of Earth and Planetary Sciences, University of New Mexico, Albuquerque, New Mexico, United States of America; 5 Department of Molecular Genetics and Microbiology, University of New Mexico, Albuquerque, New Mexico, United States of America; McGill University, Canada

## Abstract

**Background:**

Pathogen-specific metabolic pathways may be detected by breath tests based on introduction of stable isotopically-labeled substrates and detection of labeled products in exhaled breath using portable infrared spectrometers.

**Methodology/Principal Findings:**

We tested whether mycobacterial urease activity could be utilized in such a breath test format as the basis of a novel biomarker and diagnostic for pulmonary TB. Sensitized New-Zealand White Rabbits underwent bronchoscopic infection with either *Mycobacterium bovis* or *Mycobacterium tuberculosis*. Rabbits were treated with 25 mg/kg of isoniazid (INH) approximately 2 months after infection when significant cavitary lung pathology was present. [^13^C] urea was instilled directly into the lungs of intubated rabbits at selected time points, exhaled air samples analyzed, and the kinetics of δ^13^CO_2_ formation were determined. Samples obtained prior to inoculation served as control samples for background ^13^CO_2_ conversion in the rabbit model. ^13^CO_2_, from metabolic conversion of [^13^C]-urea by mycobacterial urease activity, was readily detectable in the exhaled breath of infected rabbits within 15 minutes of administration. Analyses showed a rapid increase in the rate of ^13^CO_2_ formation both early in disease and prior to treatment with INH. Following INH treatment, all evaluable rabbits showed a decrease in the rate of ^13^CO_2_ formation.

**Conclusions/Significance:**

Urea breath testing may provide a useful diagnostic and biomarker assay for tuberculosis and for treatment response. Future work will test specificity for *M. tuberculosis* using lung-targeted dry powder inhalation formulations, combined with co-administering oral urease inhibitors together with a saturating oral dose of unlabeled urea, which would prevent the δ^13^CO_2_ signal from urease-positive gastrointestinal organisms.

## Introduction

After a long hiatus in the development of new drugs for the treatment of tuberculosis (TB), exciting compounds such as TMC207/ R207910, PA824 and OPC6768 are coming out of preclinical stages and entering into clinical trials, with the promise of other new compounds to follow [1]–[3]. However, it has become clear that there is a critical need for new biomarkers of the response of TB to drug therapy, that can provide surrogates of drug efficacy to guide design and conduct of these clinical trials [4]–[6]. Without such biomarkers, these trials could be longer, more complicated and costlier, thereby delaying the availability of new drugs and perhaps even discouraging trials of others. Surrogate biomarkers are especially important in Phase IIa/b design, where issues such as drug doses and regimen design are often complex, although they would be useful throughout a trial, and beyond into treatment monitoring.

The need for these new biomarkers derives from inadequacies in current endpoints. In particular, the current standard, of relapse-free survival for 2 years after treatment, makes trials long and unwieldy, although a well-justified earlier biomarker(s) might be acceptable to regulatory agencies in certain circumstances. Ideal biomarkers would be rapid and easy to use even in relatively low resource settings, and administered and read in point-of-care manner. To be useful in the design and implementation of drug trials, it should allow sensitive and dynamic monitoring of mycobacterial burden treatment throughout much (if not all) of a treatment regimen in a wide range of patient populations, providing a surrogate for the process of whole organ homogenization and determination of colony forming units (CFU) that can only be obtained in preclinical animal studies [4]. The three current biomarkers available, sputum smear positivity, early bactericidal activity (EBA) or conversion to negative sputum culture at two months, do not fulfill these requirements although they continue to be useful, with the later also accepted as a biomarker of sterilizing activity. Although immune-based surrogates are invaluable in diagnosis, there are concerns about their usefulness to dynamically observe the effects of therapy upon bacterial load, and also about their use in immuno-suppressed and pediatric populations. Furthermore, the variance in host response and uncertainties in the relationship between CFU and biomarker response, mean that these approaches are unlikely to provide the required information.

Pathogen-specific metabolic pathways may be rapidly detected by breath tests based on introduction of nontoxic isotopically-labeled substrates and detection of labeled products that are specific to microbial metabolism in exhaled breath. Detection of ^13^CO_2_ in exhaled breath is particularly attractive because the ratio of ^13^CO_2_ to ^12^CO_2_ may be readily measured using either mass spectrometry, or by portable infrared spectrometers. This approach has been employed to diagnose *Helicobacter pylori* infection and to monitor of its eradication by drug treatment [7] using bacterial urease as a biomarker, with isotope breath testing based upon giving patient an isotopically labeled urea drink and monitoring exhaled labeled CO2. The entire diagnostic process can be completed within about 15 minutes (for example using the Breathtek™ test from Otsuka) using a portable infrared detection device and stable tracer packages. *M. tuberculosis* also possesses an active urease, used in classical microbiological assays [8]–[12], and also a potential virulence factor that enhances intracellular survival by alkalinizing the microenvironment and preventing phagosome-lysosome fusion [13]–[15].

We hypothesized that an approach based upon monitoring specific mycobacterial metabolic conversion of stable-isotope-labeled substrate into labeled exhaled gas, analogous to the widely-accepted breath test for *Helicobacter pylori*, together with lung delivery of the labeled substrate, would allow the detection of *M. tuberculosis* within the lung without having to obtain sputum or other samples that may only partially report upon the total lung. Furthermore, we hypothesized that the extent of conversion of the labeled-substrate to labeled gases is dependent upon bacterial density, and so this test could report upon total bacterial load and its response to therapy, and so provide a useful biomarker. Finally, if a biomarkers predictive ability is shown in clinical trials of new drugs, it could then be used for rapid point of care monitoring, and guide treatment of TB cases, especially MDR/XDR for which the increased monitoring costs would be justified by improved treatment outcomes with fewer failures [16], [17].

In order to test our hypothesis, we used an animal model that utilizes presensitization and bronchoscopic inoculation to reliably produce pulmonary cavities in the rabbit model of tuberculosis [18]. Because this model achieves bacillary burdens that are comparable to those observed in humans with advanced pulmonary TB, it mirrors levels of mycobacterial urease in patients with TB. To drive lung specificity, the tracer, a ^13^C-urea solution was directly instilled in the airways of infected animals at various time points during infection. This models pattern of tracer distribution we would obtain using a dry powder inhalation dose delivery system, which would be the preferred ultimate embodiment for human treatment we plan. Exhaled breath specimens were collected and the ^13^CO_2_/^12^CO_2_ ratio was quantified by isotope ratio mass spectrometry over a selected interval. Approximately 8 weeks after infection, isoniazid (INH) was given daily for a 14-day interval. Breath testing was obtained at multiple time points both during and after treatment to determine if mycobacterial urease activity could be utilized as a rapid biomarker for pulmonary TB and its response to therapy.

## Methods

### Ethics Statement

All animals were maintained under protocol ID number RBO8M512 which is approved by the Institutional Animal Care and Use Committee of Johns Hopkins University.

### Microorganisms

Cultures were prepared by thawing frozen stock aliquots of *Mycobacterium bovis* (*M. bovis*) Ravenel and *M. tuberculosis* (*M. tb.*) H37Rv. Mycobacteria were grown in 7H9 Middlebrook liquid medium supplemented with oleic acid, albumin, dextrose and catalase (Becton Dickinson, Inc., Sparks, MD), 0.5% glycerol and 0.05% Tween 80.

### Urease activity

Cultures of *M. tb.* CDC 1551, *M. bovis* BCG, and two urease deficient mutants (M. tb. CDC 1551 Δ*ure*B and Δ*ure*D) were cultivated in 7H9 Middlebrook liquid medium and diluted to 10^3^ CFU/mL to test media specifications. Urease activity was measured using BD BBL™ Taxo™ Differentiation Disc Urea Kit (Becton Dickinson, Inc., Sparks, MD) Colorimetric changes were monitored after one day and three days post- urea disc introduction. Optical density measurements were undertaken at an OD550nm after 3 days of incubation with the urea discs. Specificity of urease activity was normalized to the CFU count at the time of optical density calculations (Figure S1).

### Animals

6 pathogen-free outbred New Zealand White rabbits (2.5–3.5kg) were obtained from Covance Research Products, Inc. (Denver, PA). Animals were housed in biosafety level 3 conditions in standard cages. All rabbits were sensitized every 3–4 days by administration of five subcutaneous injections of 10^7^ heat-killed *M. bovis* in incomplete Freund's adjuvant. Successful acquisition of delayed type hypersensitivity (DTH) reaction was undertaken 25 days after the last sensitization injection by the intradermal injection of 0.1 cc of old tuberculin (Synbiotics Corp, Kansas City, MO). The tuberculin reaction was read 48–72 hours later and skin fold thickness was measured in two dimensions. Results were calculated using the formula for the volume of an oval spheroid.

### Infection and Clinical Assessment

Animals were sedated with ketamine (15–25 mg/kg) and xylazine (5–10 mg/kg). Reversal of sedation was achieved with yohimbine (0.1–0.2mg/kg). Infection was administered by instillation of a liquid inoculums through a 3.0mm flexible bronchoscope Pentax FB-8V pediatric bronchoscope (Pentax Medical Company, Montvale, NJ) into the subsegmental bronchi of the right middle lobe. A bacillary suspension of 0.3cc containing 10^3^–10^4^ CFU was delivered through the insertion port of the bronchoscope. Confirmation of the amount of CFU delivered was performed by plating serial dilutions of the inoculated suspension – as described above. After infection, animals were monitored twice weekly for clinical appearance, rectal temperature and weight.

### Antimicrobials

Isoniazid (INH) was obtained and formulated for intramuscular (IM) administration. 25mg/kg of INH was administered IM daily for 2 weeks approximately eight weeks after bronchoscopic infection.

### Urea Breath Sampling

A suspension of 40–50mg of ^13^[C] labeled urea (100 mg/mL) was instilled via a small bore feeding tube introduced through a 4.0mm endotracheal tube. The feeding tube was advanced to be at the level of the carina or slightly beyond the orifice of the right mainstem bronchus. After instillation of the urea, the animals were held supine with 45 degree head elevation to enhance distribution of the fluid to the lower lung zones. At set time points (5 minutes, 10 minutes and 20 minutes) after isotopic urea instillation, a 14-French feeding tube was then introduced through the endotracheal tube to the level of the carina. A 10cc syringe attached to the feeding tube sampled air with occasional forced expiratory maneuvers being undertaken (i.e. gentle squeezing of the rib cage). Sampled air was then inserted through a 0.35micron filter into a 6.0mL red-top Vacutainer (Becton Dickinson, Franklin Lakes, NJ, USA). These air sampling procedures were undertaken prior to bacillary infection and selected time points post-infection. At each time point, baseline samples of environmental air with ambient ^13^CO_2_ and ^12^CO_2_ were obtained.

### Measurement of ^13^CO_2_ Conversion

3 ml of 0.2 micron filtered exhaled breath sample was transferred into a 10ml septa-sealed vial containing helium gas, and analyzed by gas isotope ratio mass spectrometry using a Delta^plus^XL instrument for ^13^C enrichment in headspace CO_2_. The ^13^C enrichment in CO_2_ (δ^13^CO_2_) is reported is in the delta notation, where the delta value is given in ‰ as follows:
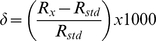
where *R* is the ratio of the abundance of the heavy to light isotope, *x* denotes the sample, and *std* is an abbreviation for standard, Vienna Pee Dee Belemnite (VPDB). Reproducibility in δ^13^C was better than 0.1‰. The values of δ^13^CO_2_ were found to increase linearly with time after urea administration, and so these values were fitted linearly, and the increase in δ^13^CO_2_ per minute evaluated.

### Necropsy

Rabbits were observed for a minimum of 50 days after infection. Euthanasia was performed if animals displayed any signs of respiratory distress and/or significant loss of weight (greater than 200g). Rabbits were euthanized with intravenous euthasol (Virbac Corporation, Fort Worth, TX). Specimens were obtains from the lungs and extrapulmonary locations. Cavity specimens, where applicable, represented the lumen contents, wall and surrounding inflammatory tissue. Extrapulmonary sites included the spleen, liver, kidney (bilateral), urine, feces, Peyer's patches in the small intestine.

### Scoring of gross pathology and cavity histopathology

Grossly visible pulmonary primary lesions in the right lower lobe and secondary lesions in the ipsilateral & contralateral lungs were scored according to their prevalence. Grossly visible extrapulmonary lesions were scored according to their prevalence and location. Tissue sections were embedded with paraffin and stained with hematoxylin and eosin. All slides were examined with a Nikon Microscope Eclipse E800 (Nikon Instruments Inc., Melville, NY).

### Scoring of bacterial CFU counts

CFU counts were measured at all predetermined pulmonary and extrapulmonary locations from each infected rabbit. Tissue samples from each site were homogenized and plated on selective 7H11 agar as described above. Enumeration of CFU counts occurred on days 14, 21 and 28.

### Statistics

SigmaStat 3.0 was used to determine statistical values, using Anova and t-test, a p value of 0.05 or less indicating significance

## Results

### In vitro studies

**Figure 1:**
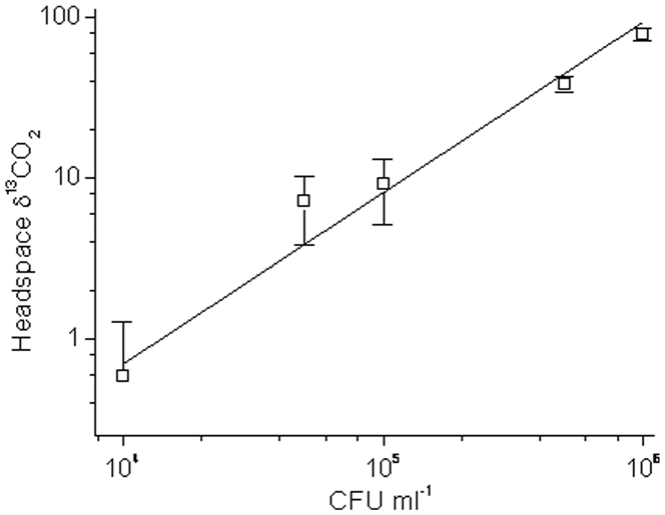
Mycobacterial urease assay by conversion of [^13^C] urea to ^13^CO_2_ reports upon CFU over a wide range. *M. bovis BCG* was incubated for 30 minutes with 5 mg ml^−1^ [^13^C]-urea and headspace gas sampled and analysed for ^13^CO_2_ enrichment (reported as δ^13^CO_2_).

The linearity of a potential biomarker with CFU count is important, and so *M. bovis BCG*, a vaccine strain of *M. tuberculosis* was incubated with ^13^C-labeled urea and headspace gas analyzed after 30 minutes for δ^13^CO_2_ (the enrichment of headspace CO_2_ with ^13^CO_2_) and results of a typical experiment shown in Figure 1. It can be seen that δ^13^CO_2_ increased in a linear manner with CFU over more than 2 orders of magnitude, and that the slopes (extent of conversion per 10^6^ CFU) for different experiments were similar (data not shown). Importantly, detection was possible at levels of 10^4^ CFU ml^−1^, significantly lower than most observed levels in sputum, which are observed in the range of 10^6^ to 10^8^ CFU ml^−1^ in EBA studies so that there is the potential for great sensitivity (17).

### Varying lung pathology following presensitization and bronchoscopic infection

**Figure 2:**
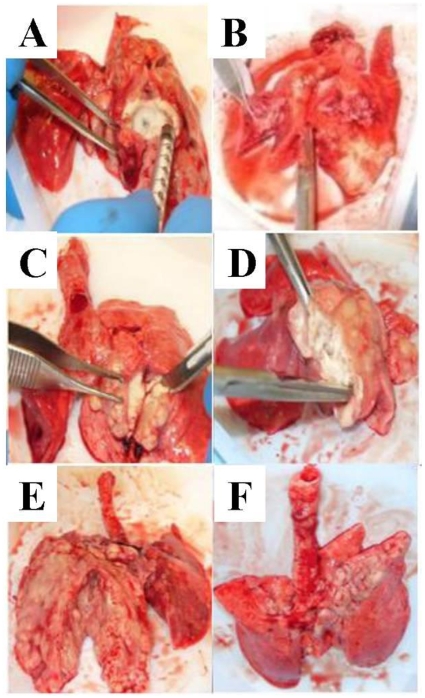
Gross pathology of selected lung specimens at necropsy. (A) *M. tuberculosis* H37Rv T1. (B) *M. tuberculosis* H37Rv T2. (C) *M. bovis* B1. (D) *M. bovis* B2. (E) *M. bovis* B3. (F) *M. bovis* B4.

Each rabbit was effectively sensitized with heat-killed *M. bovis* and all converted their tuberculin skin tests to positive prior to infection similarly to previous work (Table 1) (18). Even Rabbit B1, *which* showed the least amount of skin test reactivity, successfully generated a lung cavity. Varying gross pathology had been noted utilizing direct bronchoscopic infection into the right middle lobe (Figure 2). Both rabbits T1 and T2 infected with *M. tuberculosis* H37Rv did not develop full cavitary lesions. Rabbit T1 developed diffuse granulomatous disease that was most prominent in the initial site of infection in the right middle lobe. No signs of liquefaction or caseation were appreciated on dissection of the right middle and lower lobes. CFU counts from rabbit T1 yielded over 10^6^ bacilli from the right lung and slightly greater than 1.5 log from the contralateral side (Table S1). Rabbit T2 displayed minimal granulomatous disease with focal areas of necrosis. Necropsy of the rabbit was complicated by diffuse pulmonary hemorrhage after intubation. The lungs were grossly engorged with multiple clots appreciated in the bilateral lung vasculature. No observable CFUs were noted from plating various specimens from bilateral lung sections from rabbit T2.

**Table 1:** Disease parameters associated with rabbit infection.

Rabbit	Skin Reaction (mm^3^)‡	Days of Infection	Time Points (days) Breath Testing Obtained§
T1*	1703	96	*Day 0* (pre-infection), *Day 54* (pre-treatment), *Day 63* (0 days post-treatment)
T2*	2159	126	*Day 0* (pre-infection), *Day 62* (pre-treatment), *Day 69* (7 days into treatment), *Day 104* (33 days post-treatment), *Day 115* (44 days post-treatment), *Day 127* (56 days post-treatment)
B1†	3250	50	*Day 0* (pre-infection), *Day 44* (pre-treatment)
B2†	937	93	*Day 0* (pre-infection), *Day 47* (pre-treatment)
B3†	1524	92	*Day 0* (pre-infection), *Day 49* (pre-treatment), *Day 63* (10 days into treatment), *Day 89* (19 days post-treatment)
B4†	1434	90	*Day 0* (pre-infection), *Day 42* (pre-treatment)*Day 89* (19 days post-treatment)

* *M. tuberculosis* H37Rv.

† *M. bovis* Ravenel.

‡ Skin reaction was measured as cutaneous reactivity to Old Tuberculin 25 days after the last pre-sensitization. The tuberculin reaction was read 48–72 hours after subcutaneous injection of Old Tuberculin to confirm successful acquisition of DTH immunity.

§ Exhaled air samples were extracted at varying frequencies both prior and after treatment with INH (25mg/kg). Treatment was provided daily over a 14 day time interval. Select rabbits were administered breath testing at multiple time points during infection and after treatment.

Rabbits B1, B2, B3 and B4 were infected with *M. bovis*. All formed cavitary lesions with the exception of rabbit B4 (Figure 2). The highest total CFU counts were measured in the inner cavitary contents of B1 and B3, which yielded over 10^8^ bacilli (Figure S2). Rabbit B2 contained approximately 0.3 log unit more CFUs in the right lung as compared to liquid caseum. Contralateral dissemination to the left lung was noted in all cavitary rabbits. Rabbit B4 developed no cavitary lesion but notable granulumatous disease isolated to the bilateral upper lobes was appreciated. Specimens from bilateral sections yielded CFU only from the right lung. Thus, the animals displayed a range of pathologies, and thus their use represented a realistic test of potential success of the approach in a similar human population.

### Bacterial correlates of disease phenotype: extrapulmonary dissemination of *M. bovis* but not *M. tuberculosis*

In contrast to *M. tuberculosis* infections, *M. bovis*-infected rabbits uniquely showed bacterial dissemination to extrapulmonary organs which was consistent with our previous lung cavitation studies (Figure S3). Rabbits B1 and B3 had notable splenic CFU counts. Greater than 2.5 log units of kidney CFU counts were seen in all *M. bovis*-infected rabbits with the exception of B1. The kidney CFU counts were approximately 1 log greater than in the spleen. Grossly visible granulomas were noted on the surface of these kidneys. Extrapulmonary dissemination of *M. tuberculosis* was not observed on gross pathology or microbiologic assessment. Both the *M. tuberculosis* H37Rv strain and the *M. bovis* Ravenel strain were tested for urease activity by the urea disk assay and were found to be strongly positive (Figure S1).

### Diagnostic utility of urea breath testing in tuberculous rabbits

**Figure 3:**
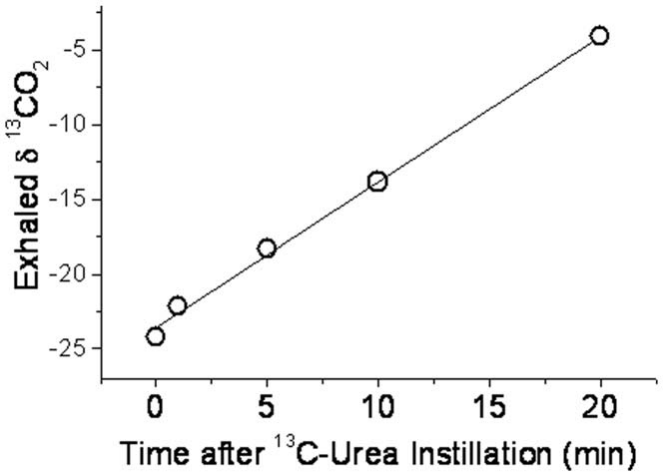
Exhaled δ^13^CO_2_ increased linearly after urea instillation. Breath samples were obtained prior to, and 1, 5, 10 and 20 minutes after urea instillation. Fig 3 shows typical data from a heavily infected rabbit.

**Figure 4:**
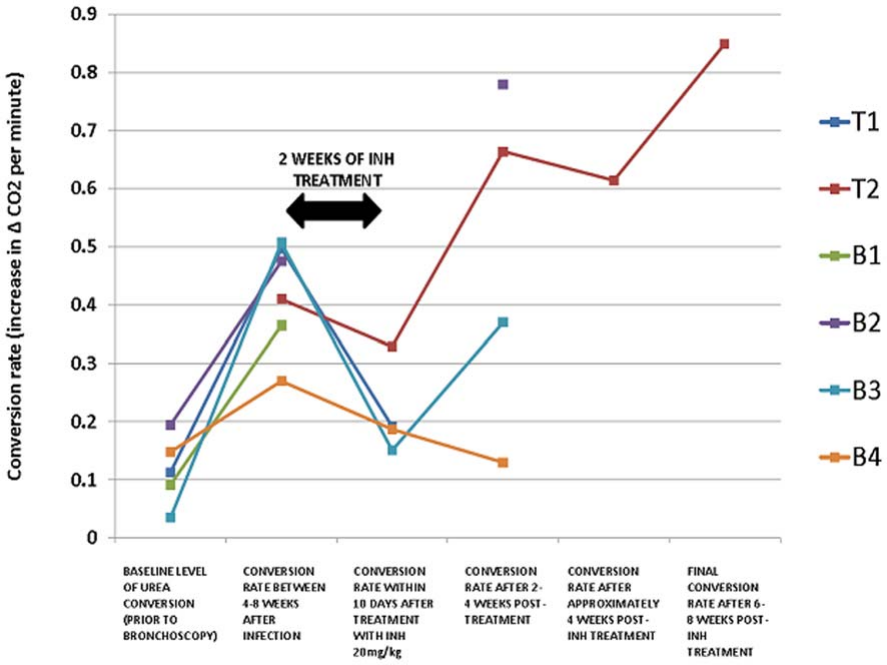
Conversion rate of [^13^C]-urea prior and after treatment with INH. Baseline level of [^13^C]-urea conversion was obtained on all animals prior to bronchoscopic infection. Urea conversion rates were obtained at a single time point after 4–8 weeks post-infection. INH 20mg/kg daily for a 2-week interval was initiated after 8 weeks from the time of infection. Conversion rates were ascertained either during or within 10 days of treatment cessation. Urease conversion were lastly determined at single time points after 2–4 weeks and 6–8 post-treatment in selected rabbits.

All rabbits had urea breath testing performed prior to bronchoscopic infection, and some detectable enzymatic conversion of [^13^C]-urea was noted at baseline. The increase in δ^13^CO_2_ after urea administration was observed to follow approximately linear kinetics over 20 minutes (typical example of a post infection case in Figure 3 and so these were fitted to provide the rate of increase in δ^13^CO_2_ minute^−1^.The δ^13^CO_2_ minute^−1^ conversion rate ranged from 0.1 to 0.2 [per mil units/minute] (Figure 4). Increasing UBT signals were observed in all evaluable rabbits (with presumed lower CFU count), during the range of days that breath testing was initially undertaken after infection, 42–62 days (Figure 4). All conversion rates were greater than 0.2 [per mil units/minute] post-infection. The rabbits with the most prominent lung pathology showed the greatest detectable δ^13^CO_2_ signal. Rabbit B4 had the least amount of grossly observable pathology on necropsy (bilateral upper lobe granulomas) and the lowest conversion rate. Rabbit B4 had approximately 3 log CFU/ml appreciable in the right lung and may serve as the possible basis of the lower CFU limit of detection (Figure 2). Rabbit T1, with numerous bilateral granulomas and no cavitary disease on necropsy, had the highest detectable δ^13^CO_2_ post-infection.

### Urea breath testing as a treatment response marker in tuberculous rabbits

INH (25mg/kg) was provided daily for a 14-day interval to all rabbits but B1. Rabbit B1 was euthanized prior to treatment due to significant respiratory distress observed prior to the treatment time period. [^13^C]-urea conversion rates were tested during treatment for all rabbits with the exception of B1 and B2 (Table 1). During and/or within 2 weeks post-treatment, a decrease in labeled urea conversion was appreciated in all tested animals. Rabbit T1 had the steepest decline in detectable δ^13^CO_2_.

**Figure 5:**
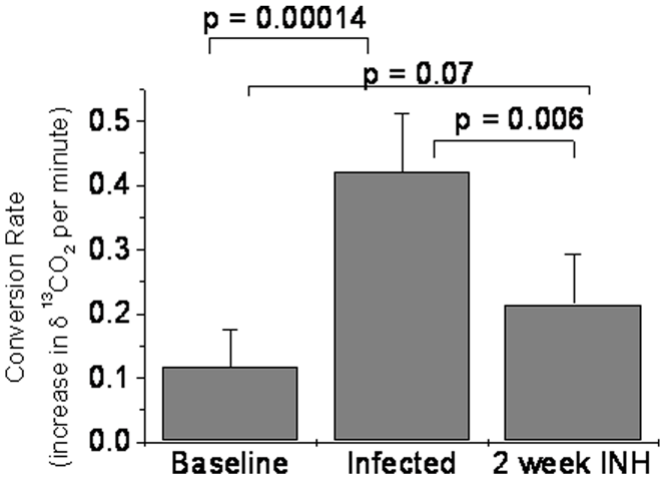
Mean conversion rates of [^13^C]-urea per minute among *M. tuberculosis* H37Rv and *M. bovis*-infected rabbits. Repeated measurements had occurred of each breath test sample. Significant conversion rate differences are noted after both infection and treatment with 25mg/kg of INH. Rates were obtained prior to infection, 4–8 weeks after bronchoscopic infection and within two weeks post-INH treatment.

After 2 weeks post-treatment, a rise in δ^13^CO_2_ was noted in rabbit T2. Though no observable CFU counts were noted on necropsy, minimal gross pathology was appreciated. This continued rise in urease activity was noted after 8 weeks post-infection in rabbit T2. It Rabbits B3 and B4 had a notable decline in the conversion rate after 2 weeks post-INH treatment. Rabbit B3 showed a detectable δ^13^CO_2_ after 4 weeks post-treatment likely due to the high bacillary burden in the developed lung cavity. Rabbit B4 demonstrated solely upper lung lesions and a continued decline in the conversion rate was appreciated 4 weeks after treatment cessation. Rabbit B2 did not undergo urease detection 2 weeks post-treatment. However, testing performed 4 weeks post-treatment yielded a continued elevation in detectable δ^13^CO_2_. The continued rise in urease conversion in rabbit B2, as in rabbit B3, may be attributable to the formation of a significant cavitary lesion with a high bacillary burden (Figure 2). Only rabbit T2 had time points distal to 4 weeks post-treatment. Cumulative data of all animals noted post-infection demonstrated a significant increase in the mean δ^13^CO_2_ conversion rate (Figure 5), that was significantly decreased after treatment.

## Discussion

The results of this study suggest that delivery of [^13^C]-urea into the lungs of rabbits infected with urease-producing mycobacteria could be used as a diagnostic modality to estimate lung burdens of disease and their response to therapy. Both *M. tuberculosis* and *M. bovis* infected rabbits have a significant rise in the rate of δ^13^CO_2_ formation prior to treatment that was decreased after INH therapy. Although the rise in urea conversion was most prominently observed in rabbits with cavitary or diffuse lung pathology, it was notable even in rabbits with the greatest amount of localized disease (infection in the right middle lobe as evident in rabbits T1 and T2) so that the approach would seem applicable broadly. The improved patterns of lung delivery possible with dry powder inhalers could improve upon this aspect yet further.

Urease is present in most pathogenic mycobacteria, including *M. tuberculosis* and *M. bovis* [19]. The presence of urease in mycobacteria was first noted by Söhngen in 1913 and confirmed by Corper and Sweany in 1918 [20]–[22]. The testing was based on the enzyme's ability to degrade urea into CO_2_ and NH_3_, the latter of which served as the foundation for bacterial classification. Toda et al. refined the classification of mycobacteria by urease activity through the development of a rapid colorimetric broth technique [8]. This was followed years later with further development of rapid urease-based testing that involving urea-embedded test discs and modified BACTEC radiometric instrumentation [11], [12]. Since the last major publications on urease-based diagnostics in the 1970s, no further work dedicated to this subject matter has been noted after an extensive review of the literature. Our work stems from fusing this classical approach of mycobacterial classification and more recent urease-based testing for the diagnosis of *H. pylori*.

Urea breath testing (UBT) for *H. pylori* involves the oral intake of [^14^C]- or [^13^C]-urea and assessing the δ^14^CO_2_ or δ^13^CO_2_ through exhaled breath specimens. This sensitive diagnostic is now commonly utilized as a rapid test of *H. pylori* infection [7], [22]. Our model contains an important modification of *H. pylori* UBT in that instead of oral delivery of the labeled urea, we used direct intrapulmonary administration of the non-radioactive tracer, delivering the testing agent to the area of interest. No adverse respiratory events were noted after instillation of the urea compound. Lung-targeted delivery of the [^13^C]-urea via dry powder inhalers will represent our delivery for future human studies, in which more efficient diffuse lung deposition of the testing agent will be required [23].

Our model potentially offers a path toward a novel point-of-care diagnostic for TB and TB treatment response. Point-of-care diagnostics are desperately needed in high-burden settings in order to reduce delays in disease detection and to accelerate the recognition of treatment failure suggestive of drug-resistant tuberculosis [24]. Delays in diagnosis, which characterizes several modern TB diagnostics, may be directly attributable to the inability to control the epidemic [25]–[27]. The conversion rate per minute shows the test may rapidly and reliably detect pulmonary tuberculosis associated with a variety of pathologic presentations. The animals in our study displayed not only cavitary lesions, but also diffuse granulomatous disease and isolated upper lobe granulomas. Despite the variability in the tissue-based disease, each animal displayed a significant rise in δ^13^CO_2_ after infection. The developed test fulfills the criteria for effective point-of-care diagnostic which requires an easily instituted, cost-effective and rapidly applied technology. Isotopic gas ratio analysis may now be conducted with compact infrared spectrophotometers such as POCone™ (Otsuka Pharmaceutical Co., Ltd., Tokyo, Japan). Exhaled air may be simply obtained through one-way valve bags and analyzed in this device.

Our model also provides a novel biomarker for TB drug therapy trials. Biomarkers are actively being sought to improve therapeutic strategies and validate novel TB drug and vaccine candidates [4], [6]. INH was given daily for 2 weeks to 5 rabbits of which 4 had time points taken after the medication period. All four rabbits had a decline in their δ^13^CO_2_ conversion rate upon testing just prior to 2 weeks post-treatment. Continued decline in conversion rates were noted in rabbit B4 which demonstrated solely bilateral upper lobe granulomatous disease. Treatment of such minimal disease with 2 weeks of INH was demonstrated with the continued decline in conversion rates. Adaptation of this test to humans might allow for serial monitoring of a biomarker of lung CFU during treatment and offer valuable early information on whether bacterial burdens are responding to antimicrobial therapy. Breath testing may allow for actionable information within the time frame of minutes to determine if TB bacilli are sensitive to an employed drug regimen. As opposed to currently utilized sputum analyses or molecular-based assays being used for drug susceptibility, urea breath testing for *M. tuberculosis* would allow for a rapid acknowledgement of treatment efficacy and need for treatment modifications for drug-resistant TB [28], [29].

It is worth noting that the approach detailed here has the potential to be much more rapid and easy to apply than classical EBA studies to determine the usefulness of a chemotherapeutic agent, as 3 to 4 weeks cultures for CFU are not required. The potential also exists to avoid some known drawbacks of EBA- the need for extensive sputum, collection, the limited sampling of site of sputum production (as opposed to the entire lung), and the potential for bacterial and/or fungal contamination of cultures [30]. EBA studies assess the early phase of TB treatment, in which a drug's ability to kill metabolically active bacilli is being evaluated [31]. However, the assessment of the latter sterilization phase of treatment has been one of great debate. Extended EBA studies (days 2 to 14 of treatment) have unfortunately not proven itself as a significant surrogate marker for the detection of sterilizing activity [32], [33]. Currently, the traditional 2 month culture conversion is the most commonly utilized method being employed as a marker of sterilization [34]. Our test successfully detected the bactericidal activity of INH which was active against the numerous actively replicating bacilli in our cavitary rabbits over a 14-day time interval. Future experiments to determine the utility of this approach would include the use of sterilizing drugs (i.e. rifampin, pyrazinamide, etc.) over a designated time interval so as to also determine the value of urea breath testing as a marker of sterilization activity.

Our study was limited by the possible lack of specificity, the positive baseline signal, resulting from other urease-producing microbial species (such as select Pseudomonas, Klebsiella, non-tuberculous mycobacteria and *H. pylori*) [35], [36]. This organism is of particular importance for future translational work given that approximately 50% of the world's population is infected and the highest rates are in developing nations [37]. Most *Helicobacter* species are rapidly cleared by rifampin treatment and therefore serial UBT measurements in rifampin-treated patients (such as in early drug trials in already-treated patients) might not show long-term false-positivity due to endogenous *Helicobacter* carriage [38], [39].

However, broader adoption will require solutions to this potential confounder. It would be possible to combine inhaled labeled urea in humans (to drive lung specificity of tracer delivery) with an oral therapy that includes inhibitors of Helicobacter urease, such as bismuth salts (i.e. Pepto-Bismol®) or proton pump inhibitors or lithostat to suppress GI urease activity and possible confounding of data [40], [41]. An additional approach would be to co-administer a urea drink (similar to the Breathtek approach for *H. pylori*) that is not enriched in ^13^C, but rather has this at normal abundance: therefore any CO_2_ produced by gut organisms would not be enriched in ^13^CO_2_ and thus not confound the signal from the lungs. Finally, in humans, measurements could be made rapidly after urea inhalation, before sufficient time for any urea to reach the gut, converted to CO_2_ and then exhaled. Such approaches would be most easily tested and adopted in clinical trials of new drugs, where support infrastructure and patient characterization would be much higher than in the field, and success there would allow for broader adoption.

In summary, this study describes a novel use of [^13^C]-urea breath testing as a diagnostic and treatment biomarker for TB using lung delivery of tracer. A significant rise in δ^13^CO_2_ was noted after 6 weeks post-infection in all rabbits which displayed differing pulmonary pathologies. The reversal in the rate of conversion after two weeks of daily therapy with INH was also appreciated in selected rabbits within 2 weeks post-treatment. This experimental approach may have important implications for future development of point-of-care diagnostics, treatment monitoring, and testing of new TB therapeutics.

## Supporting Information

Table S1 Mean log pulmonary, spleen and kidney CFU counts at necropsy for all rabbits. Multiple samples of the right lung (the site of infection), left (contralateral) lung, cavity wall, caseous cavitary material, spleen and kidney tissue were removed from areas with the greatest discernable gross pathology. The log CFU count/gram of tissue was determined after tissue homogenization and plating dilutions. *M. bovis* infected rabbits had displayed the greatest intrapulmonary and extrapulmonary pathology.
(0.03 MB DOC)

Figure S1 Urease activity of select Mycobacterium species. (A) Differentiation of observed urease activity was undertaken of *M. bovis* BCG, *M. tb.* CDC 1551, and two urease deficient mutants (*M. tb.* CDC 1551 Δ*ureB* and Δ*ureD*), Colorimetric changes were visually observed after one and three days post inoculation with a urea-embedded disc. (B) Optical density measurements were undertaken after 3 days of incubation with the urea discs and specificity of urease activity was normalized to CFU counts.
(0.18 MB TIF)

Figure S2 Mean pulmonary CFU counts at necropsy for evaluable *M. bovis*-infected rabbits. Multiple samples of the right (the site of infection) and left (contralateral) lung tissue were removed from areas with the greatest discernable gross pathology. Samples from the cavity wall and luminal caseous contents material were also obtained. The log CFU count/gram of tissue was determined after tissue homogenization and plating dilutions. The graph displays solely evaluable rabbits with detectable CFUs in all lung tissues. Additional information is in Table S1.
(0.07 MB TIF)

Figure S3 Mean spleen and kidney CFU counts at necropsy. Multiple samples of kidneys and spleens were removed from tissues with the greatest discernable gross pathology. The log CFU count/gram of tissue was determined after tissue homogenization and plating dilutions. Additional information is in Table S1. As noted in previous published experiments, *M. tuberculosis* H37Rv demonstrated no evidence of extrapulmonary dissemination as compared to *M. bovis* ^24^.
(0.05 MB TIF)
