# Supplementary material for: 13[C]-Urea Breath Test as a Novel Point-of-Care Biomarker for Tuberculosis Treatment and Diagnosis
Source: PLoS One. 2010 Aug 27;5(8):e12451. doi: 10.1371/journal.pone.0012451 (PMC2929202; doi:10.1371/journal.pone.0012451)
Supplement: Table S1 — Mean log pulmonary, spleen and kidney CFU counts at necropsy for all rabbits. Multiple samples of the right lung (the site of infection), left (contralateral) lung, cavity wall, caseous cavitary material, spleen and kidney tissue were removed from areas with the greatest discernable gross pathology. The log CFU count/gram of tissue was determined after tissue homogenization and plating dilutions. M. bovis infected rabbits had displayed the greatest intrapulmonary and extrapulmonary pathology. (0.03 MB DOC) [file pone.0012451.s001.doc]

**TABLE S1. Mean log pulmonary, spleen and kidney CFU counts at necropsy for all rabbits.** Multiple samples of the right lung (the site of infection), left (contralateral) lung, cavity wall, caseous cavitary material, spleen and kidney tissue were removed from areas with the greatest discernable gross pathology. The log CFU count/gram of tissue was determined after tissue homogenization and plating dilutions. *M. bovis* infected rabbits had displayed the greatest intrapulmonary and extrapulmonary pathology.

|  | **Right Lung (Site of Infection)** | **Left Lung (Contralateral Site of Infection)** | **Cavity Wall and Caseous Material** | **Spleen** | **Kidney** |
| --- | --- | --- | --- | --- | --- |
| **T1** | **5.31** | **3.57** | **0** | **0** | **0** |
| **T2** | **0** | **0** | **0** | **0** | **0** |
| **B1** | **4.8** | **4.9** | **8.6** | **2.26** | **0** |
| **B2** | **4.62** | **4.03** | **4.39** | **0** | **2.75** |
| **B3** | **5.91** | **4.88** | **8.09** | **2.13** | **2.67** |
| **B4** | **4.04** | **0** | **0** | **0** | **2.62** |
