# Supplementary figures and images for: 13[C]-Urea Breath Test as a Novel Point-of-Care Biomarker for Tuberculosis Treatment and Diagnosis
Source: PLoS One. 2010 Aug 27;5(8):e12451. doi: 10.1371/journal.pone.0012451 (PMC2929202; doi:10.1371/journal.pone.0012451)

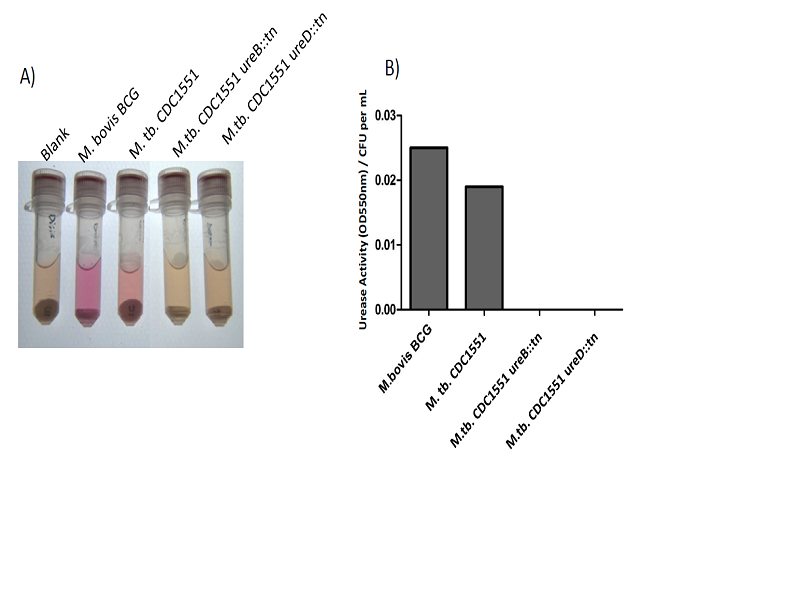

Supplement: Figure S1 — Urease activity of select Mycobacterium species. (A) Differentiation of observed urease activity was undertaken of M. bovis BCG, M. tb. CDC 1551, and two urease deficient mutants (M. tb. CDC 1551 ΔureB and ΔureD), Colorimetric changes were visually observed after one and three days post inoculation with a urea-embedded disc. (B) Optical density measurements were undertaken after 3 days of incubation with the urea discs and specificity of urease activity was normalized to CFU counts. (0.18 MB TIF) [file pone.0012451.s002.tif]

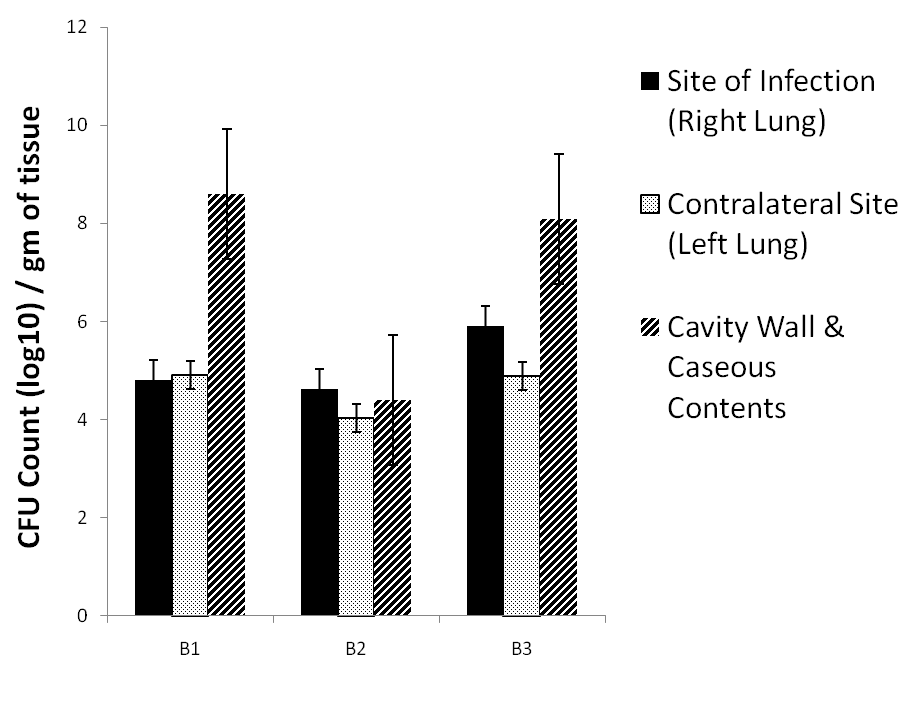

Supplement: Figure S2 — Mean pulmonary CFU counts at necropsy for evaluable M. bovis-infected rabbits. Multiple samples of the right (the site of infection) and left (contralateral) lung tissue were removed from areas with the greatest discernable gross pathology. Samples from the cavity wall and luminal caseous contents material were also obtained. The log CFU count/gram of tissue was determined after tissue homogenization and plating dilutions. The graph displays solely evaluable rabbits with detectable CFUs in all lung tissues. Additional information is in Table S1. (0.07 MB TIF) [file pone.0012451.s003.tif]

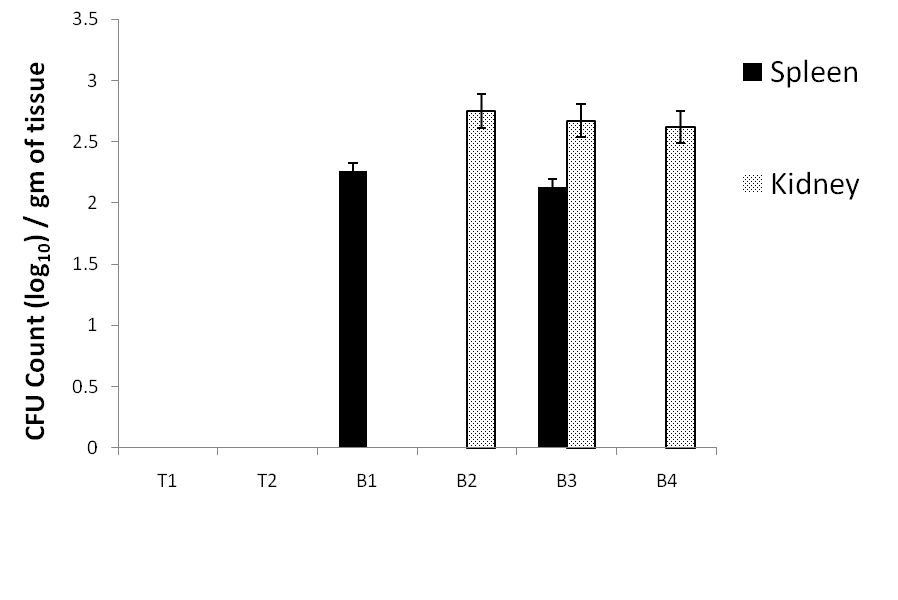

Supplement: Figure S3 — Mean spleen and kidney CFU counts at necropsy. Multiple samples of kidneys and spleens were removed from tissues with the greatest discernable gross pathology. The log CFU count/gram of tissue was determined after tissue homogenization and plating dilutions. Additional information is in Table S1. As noted in previous published experiments, M. tuberculosis H37Rv demonstrated no evidence of extrapulmonary dissemination as compared to M. bovis 24. (0.05 MB TIF) [file pone.0012451.s004.tif]
